# Protective Role of Rho Guanosine Diphosphate Dissociation Inhibitor, Ly-GDI, in Pulmonary Alveolitis

**DOI:** 10.1371/journal.pone.0140804

**Published:** 2015-10-15

**Authors:** Chunguang Yan, Ximo Wang, Yanlan Liu, Raja-Elie Abdulnour, Min Wu, Hongwei Gao

**Affiliations:** 1 Department of Anesthesiology, Perioperative&Pain Medicine, Brigham and Women's Hospital, Harvard Medical School, Boston, Massachusetts, United States of America; 2 Department of Basic Sciences, University of North Dakota, Grand Forks, North Dakota, United States of America; 3 Department of Surgery, Tianjin Nankai Hospital, Tianjin, China; 4 Pulmonary and Critical Care Medicine, Brigham and Women's Hospital, Harvard Medical School, Boston, Massachusetts, United States of America; Virginia Polytechnic Institute and State University, UNITED STATES

## Abstract

Growing evidences indicate that Ly-GDI, an inhibitory protein of Rho GTPases, plays an essential role in regulating actin cytoskeletal alteration which is indispensible for the process such as phagocytosis. However, the role of Ly-GDI in inflammation remains largely unknown. In the current study, we found that Ly-GDI expression was significantly decreased in the IgG immune complex-injured lungs. To determine if Ly-GDI might regulate the lung inflammatory response, we constructed adenovirus vectors that could mediate ectopic expression of Ly-GDI (Adeno-Ly-GDI). *In vivo* mouse lung expression of Ly-GDI resulted in a significant attenuation of IgG immune complex-induced lung injury, which was due to the decreased pulmonary permeability and lung inflammatory cells, especially neutrophil accumulation. Upon IgG immune complex deposition, mice with Ly-GDI over-expression in the lungs produced significant less inflammatory mediators (TNF-α, IL-6, MCP-1, and MIP-1α) in bronchoalveolar lavage fluid when compared control mice receiving airway injection of Adeno-GFP. Mechanically, IgG immune complex-induced NF-κB activity was markedly suppressed by Ly-GDI in both alveolar macrophages and lungs as measured by luciferase assay and electrophoretic mobility shift assay. These findings suggest that Ly-GDI is a critical regulator of inflammatory injury after deposition of IgG immune complexes and that it negatively regulates the lung NF-κB activity.

## Introduction

Ly-GDI, also known as D4-GDI, RhoGDI2, GDID4 or RhoGDIB, is a member of Rho guanidine dissociation inhibitor (RhoGDI) family that is composed of three members-RhoGDIα, RhoGDI2, and RhoGDI3 [1, 2]. RhoGDI could keep the small signal proteins, RhoGTPases, in their cytosolic inactive state by interacting with the RhoGTPases’ isoprenyl motif located in C-terminal hydrophobic pocket and suppressing dissociation of bound GDP from RhoGTPases [1–3]. When dissociated from RhoGDI and GDP, RhoGTPases become active form by binding to GTP and relocate to plasma membrane through the C-terminal isoprenyl motif [1, 4]. *In vitro* studies have demonstrated that Cdc42, RhoA, and Rac might be potential substrates of Ly-GDI [5]. The roles of RhoGDI in processes involved in cytoskeletal changes have been investigated. For example, ectopic expression of Ly-GDI blocks the cytoskeletal change, which might results in disruption of Rac-stimulated phagocytosis [6]. In T cells, Ly-GDI functions cooperatively with the GDP/GTP exchange factors (GEF), Vav1, as signal transducers in the T cell receptor (TCR) pathway, which lead to the cytoskeletal reorganization required for the immunological synapse formation [7]. Consistent with these results, a recent study finds that Ly-GDI inhibits Fcγ receptor (FcγR)-mediated phagocytosis by suppressing association of Rac with plasma membrane in human monocytes [1]. However, the impact of Ly-GDI on inflammation including the FcγR activation-induced inflammatory responses remains unknown.

IgG immune complex (IC)-induced activation of immune cells could lead to a variety of autoimmune diseases including immune thrombocytopenia, systemic lupus erythematosus, extrinsic allergic alveolitis, and rheumatoid arthritis [8–11]. IgG IC exerts its inflammatory effect by association of its Fc portion with the FcγRs. FcγRs are also involved in antibody dependent control of certain bacterial and viral infections in human and mice [12]. Several mechanisms including antibody-dependent cell-mediated cytotoxicity (ADCC), phaocytosis, and release of inflammatory mediators are involved in the pathogen clearance mediated by the interaction between pathogen-bound IgG and FcγRs [9, 13]. IgG IC-induced acute lung injury (ALI) in rodent has been used to investigate the molecular mechanisms involved in FcγR-mediated inflammatory reactions *in vivo* [14–16]. In this model, airway depositionof IgG IC stimulates alveolar macrophage to synthesize and release early response cytokines, such as TNF-α [17, 18]. The early responsive cytokines then induce various chemokine production, and the subsequent formation of chemoattractant gradients that accelerate transmigration of neutrophils from vascular vessels into lungs [19]. Activated macrophages and neutrophils release toxic reactive oxygen species (ROS) and proteases into lungs, leading to damages of pulmonary parenchyma [20] [21, 22]. The end results are elevated pulmonary microvascular permeability, intrapulmonary hemorrhage, and accumulation of protein rich edema fluid and fibrin. However, the signaling pathways that counter-regulate the inflammatory responses in IgG IC-injured lung remain largely unknown. In the current study, we evaluate the effect of Ly-GDI on IgG IC-induced ALI. By using adenovirus-mediated ectopic expression of Ly-GDI *in vivo* and luciferase assay *in vitro*, we demonstrate, for the first time, that Ly-GDI plays an important regulatory role in acute lung inflammatory responses and injury.

## Materials and Methods

### Cells and Reagents

MH-S cells (CRL-2019^™^) and HEK 293 cells (CRL-1573^™^) were obtained from American Type Culture Collection (Manassas, VA). MH-S cell were cultured in RPMI 1640 medium supplemented with 10% Fetal Bovine Serum (FBS), 2 mM L-glutamine, 100 units/ml penicillin, and 0.01 M Hepes. HEK 293 cells were cultured in Eagle's Minimum Essential Medium containing 10% FBS, 2 mM L-glutamine, and 100 units/ml penicillin. They were maintained in a humidified incubator at 37°C with 5% CO_2_. ELISA kits for mouse IL-6, TNF-α, C5a, MCP-1, MIP-2, MIP-1α, MIP-1β, and KC were purchased from R&D Systems (Minneapolis, MN). BSA and Rabbit anti-BSA IgG were obtained from Sigma-Aldrich (St. Louis, MO), and ICN Biomedicals, Inc. (Solon, OH), respectively.

### IgG IC-Induced Acute Lung Injury

All procedures involving mice were approved by the Animal Care and Use Committee of Harvard Medical School (approval number 04584) which accepts as mandatory the PHS Policy on Humane Care and Use of Laboratory Animals by Awardee Institutions and NIH Principles for the Utilization and Care of Vertebrate Animals Testing, Research, and Training. Eight-week old specific pathogen-free male C57BL/6 mice were obtained from Jackson Laboratories (Bar Harbor, ME). Mice were anesthetized intraperitoneally with ketamine HCl (100 μg/g), and IgG immune complexes-mediated acute lung injury was induced as described previously [21]. Briefly, mice received intratracheal injection of 0.24 mg rabbit anti-BSA IgG in a volume of 40 μl phosphate buffered (pH 7.4) saline (PBS). Immediately after airway administration of anti-BSA, 1 mg BSA dissolved in 200 μl PBS was injected intravenously. Negative control mice received airway injection of anti-BSA. Unless otherwise indicated, 4 h after IgG IC administration, mice were exsanguinated, the pulmonary circulation flushed via the pulmonary artery with 1 ml PBS and the lungs surgically dissected. Lungs were immediately frozen in liquid nitrogen.

### Expression Vectors and Promoter Reporters

Full-length mouse Ly-GDI cDNA was amplified from total RNA of the mouse lung using reverse transcription-PCR (Invitrogen, Carlsbad, CA), sequenced, and inserted into pcDNA3.1(+) (Invitrogen, Carlsbad, CA). Recombinant adenovirus containing mouse Ly-GDI (Adeno-Ly-GDI) was constructed by using BD Adeno-XTM Expression System 1 (BD Biosciences, Palo Alto, CA). To generate the virus, Adeno-Ly-GDI was digested with PacI and transfected to HEK-293 cells according to the manufacturer's instructions. Recombinant adenoviruses were purified by BD Adeno-X virus purification kit (BD Biosciences, Palo Alto, CA). The viral stocks were tittered using Adeno-X Rapid Titer Kit (D Biosciences, Palo Alto, CA). The mouse TNF-α and IL-6 luciferase constructs were kindly provided by Richard C. Schwartz (Michigan State University). NF-κB promoter reporter was obtained from Promega.

### Intratracheal Injection of Adenovirus

50 μl of Adeno-GFP (BD Biosciences) or Adeno-Ly-GDI (5×10^8^ pfu/mouse) was injected into mice through airway. 3 days later, lungs were harvested to determine expression of Ly-GDI, or mice were used for IgG IC-stimulated acute lung inflammation.

### Bronchial Alveolar Lavage (BAL) Fluid Collection, Total and Differential White Blood Cell Counts, and Chemokine/Cytokine ELISAs

4 hours after initiation of the acute lung injury, the thorax was opened and 1 ml of ice-cold, sterile PBS was instilled into the lung via a tracheal incision. The recovered lavage fluid (BAL) was centrifuged at 450 x g for 6 minutes and the cell-free supernatants were stored at -20°C. Cell pellets were resuspended in 1 ml of Hanks balanced salt solution (HBSS) containing 0.5% bovine serum albumin (BSA) and differential cell analyses were performed by Diff-Quik-stained cytospin preparations (Dade, Duedingen, Switzerland) counting a total of 300 cells per slide in randomly selected high-powered fields. The supernatant was used for chemokine and cytokine measurements by sandwich ELISA.

### Myeloperoxidase (MPO) Activity

Mice were scarified, and lungs were lavaged and perfused *via* the right ventricle with 1 ml of PBS. MPO activity was assessed as described previously [15, 16].

### Permeability Index

Fours hours after intratracheally administration of IgG IC, mice were exsanguinated, and the thorax was opened. 1 ml of ice-cold PBS was injected *via* an intratracheal cannula. Cell-free supernatants were obtained by centrifuging bronchoalveolar lavage (BAL) fluids at 3000 rpm for 5 min. Mouse BSA level in BAL fluids was determined by ELISA (Bethyl Laboratories, Montgomery, TX), and the permeability index was expressed as the ratio of the albumin in the IgG IC-injured lungs versus that in the control-treated lungs of same type of mice.

### Histological Assay

Fours hours after airway deposition of IgG IC, 0.6 ml of 10% buffered (pH 7.2) formalin was injected intratracheally. The lungs were fixed in a 10% buffered formalin solution for morphological assay by tissue sectioning and staining with hematoxylin and eosin (H&E).

### Luciferase Assay

Transient transfections were performed with 2×10^5^ cells plated in 12-well plates by using 0.5 μg of DNA and 1.5 μl of Fugene^®^6 Transfection Reagent in 48.5 μl of Opti-MEM I medium (Invitrogen, CA). 48 h after transfection, the cells were either incubated with or without 100 μg/ml IgG IC for 4 h. Cell lysates were used to measure luciferase activity by using the Dual-Luciferase Reporter Assay System (Promega, WI).

### Electrophoretic Mobility Shift Assay (EMSA)

Nuclear extracts of whole lung tissues were prepared as described previously [21, 22]. Briefly, frozen lungs were homogenized in 0.6% (v/v) Nonidet P-40, 150 mM NaCl, 10 mM HEPES (pH 7.9), 1 mM EDTA, 0.5 mM phenylmethylsulfonyl fluoride, 2.5 μg/ml leupeptin, 5 μg/ml antipain, and 5 μg/ml aprotinin. The homogenate was incubated on ice for 5 min and then centrifuged for 5 min at 5000 rpm at 4°C. Proteins were extracted from the pelleted nuclei by incubation at 4°C with solution B (420 mM NaCl, 20 mM HEPES (pH 7.9), 1.2 mM MgCl_2_, 0.2 mM EDTA, 25% (v/v) glycerol, 0.5 mM dithiothreitol, 0.5 mM phenylmethylsulfonyl fluoride, 2.5 μg/ml leupeptin, 5 μg/ml antipain, and 5 μg/ml aprotinin). Nuclear debris was pelleted by centrifugation at 5000 rpm for 5 min at 4°C, and the supernatant extract was collected and stored at -80°C. Protein concentrations were determined by Bio Rad protein assay using BSA as a reference standard (Pierce Co.). The EMSA probes were double-stranded oligonucleotides containing a NF-κB consensus oligonucleotide (AGTTGAGGGGACTTTCCCAGGC, Promega, Madison, WI). NF-κB probes were labeled with γ [^32^P] ATP (3,000 Ci/mmol at 10 mCi/ml, GE Healthcare, Piscataway, NJ). DNA binding reactions were performed at room temperature in a 25 μl reaction mixture containing 12 μg of nuclear extract and 5 μl of 5× binding buffer (20% (w/v) Ficoll, 50 mM HEPES pH 7.9, 5 mM EDTA, 5 mM dithiothreitol). The remainder of the reaction mixture contained KCl at a final concentration of 50 mM, Nonidet P-40 at a final concentration of 0.1%, 1 μg of poly (dI-dC), 200 pg of probe (unless otherwise noted), bromphenol blue at a final concentration of 0.06% (w/v), and water to volume of 25 μl. Samples were electrophoresed through 5.5% polyacrylamide gels in 1× TBE (90 mM Tris base, 90 mM boric acid, 0.5 mM EDTA) at 190 V for approximately 3.5 h, dried under vacuum, and exposed to X-ray film.

### RNA Isolation and Detection of mRNA by Real-Time PCR

Total RNAs were extracted from mouse lungs with Trizol (Invitrogen, CA) according to the manufacturer’s procedure. After isolation, 2 μg of total RNA was subjected to reverse transcription by using the Superscript II RNase H^-^ Reverse Transcriptase (Invitrogen, CA). The Ly-GDI mRNA level was investigated by real time PCR. PCR was performed with primers for Ly-GDI: 5’ primer, 5’- GGA CTG GCA TGA GAG TGG AT -3’ and 3’ primer, 5’- AGG TGA GGT GGT CCT GTT TG -3’. Following reverse transcription, the cDNA was amplified and quantified using a Sequence Detection System (SDS 7300) and a PCR universal protocol as follows: AmpliTaq Gold activation at 95°C for 15 s and, annealing/extension at 60°C for 1 min. The fluorescence of the double-stranded products accumulated was monitored in real time. The relative mRNA levels were normalized to levels of GAPDH mRNA in the same sample.

### Western Blot Analysis

Mouse lungs were lysed in cold RIPA buffer. Samples containing 100 μg protein were electrophoresed in a 12% polyacrylamide gel and then transferred to a PVDF membrane. Membranes were incubated with rabbit anti-Flag antibody (Abcam, MA), goat anti-Ly-GDI antibody (Santa Cruz, CA), and rabbit anti-GAPDH antibody (Cell Signaling, MA), respectively. After 3 washes in TBST, the membranes were incubated with a 1:5,000 dilution of horseradish peroxidase-conjugated donkey anti-rabbit IgG (GE Healthcare, Piscataway, NJ). The membrane was developed by enhanced chemiluminescence technique according to the manufacturer’s protocol (Thermo Fisher Scientific, Rockford, IL).

### Statistical Analysis

All values were expressed as the mean ± S. E. M. Data sets were analyzed using Student’s *t* test or one-way ANOVA, with individual group means being compared with the Student-Newman-Keuls multiple comparison test.

## Results

### Adenovirus-Mediated Ly-GDI Expression in the Lung Attenuates IgG IC-Induced Lung Injury

**Fig 1:**
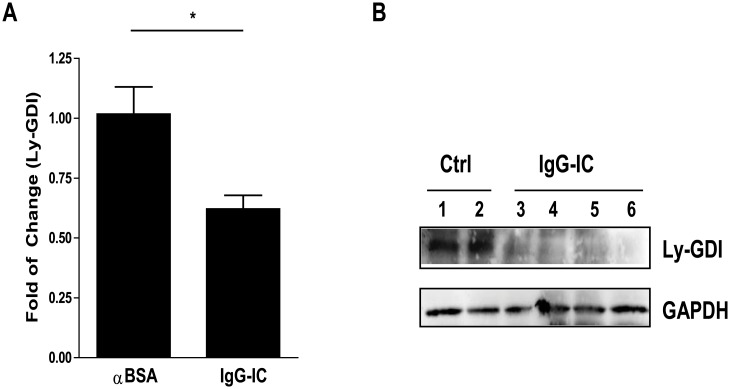
Ly-GDI expression in the lung is decreased by IgG immune complex challenge. (A) Whole lung RNAs were extracted 4 h after pulmonary deposition of IgG immune complexes, and subjected to reverse transcription. Real-time PCR was performed to examine the expression of Ly-GDI and GAPDH. Data were expressed as means ± S. E. M. N = 4 mice for each group. * indicated statistically significant difference-*p* < 0.05. (B) Total proteins obtained from control or IgG immune complex-challenged lungs were subjected to Western blot by using goat anti-Ly-GDI antibodies. The level of GAPDH was shown at the bottom as a loading control.

**Fig 2:**
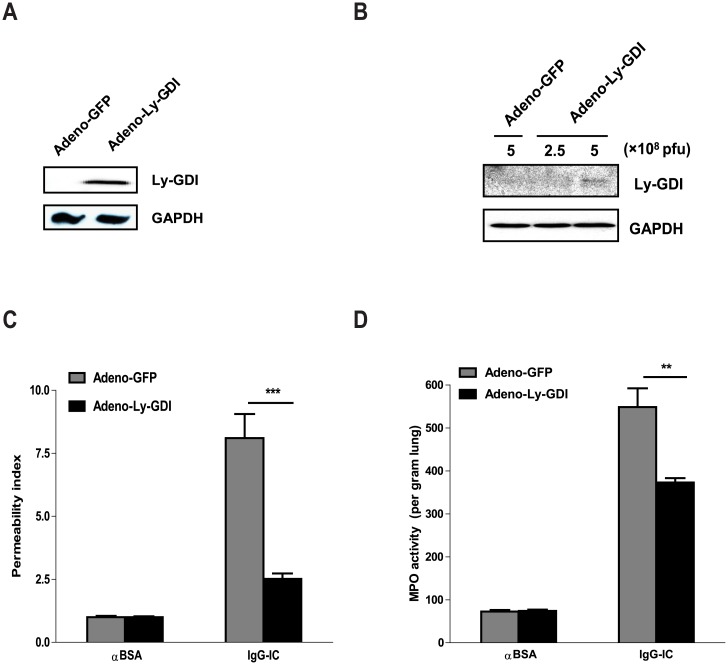
Over-expression of Ly-GDI mitigates IgG immune complex-induced acute lung injury. **(A)** HEK 293 cells were infected with Adeno-GFP and Adeno-Ly-GDI (with a Flag tag in N-terminal), respectively. When cytopathic effect became evident, the infected cells were lysed by using RIPA buffer. (**B**) Mice received intratracheal injection of Adeno-GFP or Adeno-Ly-GDI at the indicated doses. 3 days later, whole lung proteins were extracted by using RIPA buffer. Total proteins obtained from adenovirus-infected HEK 293 cells or mouse lungs were subjected to Western blot by using rabbit anti-Flag antibodies. The level of GAPDH was shown at the bottom as a loading control. (**C and D)** Adeno-GFP or Adeno-Ly-GDI was injected to mice *via* an airway instillation at a dose of 5 × 10^8^ PFU. 3 days later, the mice were subjected to IgG immune complex-induced lung injury or control treatment (αBSA only). Mouse albumin levels **(C)** in BAL fluids were measured 4 h later, and the permeability index was expressed as the ratio of the albumin in the IgG immune complex-injured lungs versus that in the control-treated lungs of same type of mice. Lung MPO activity **(D)** was evaluated, and used as a marker for pulmonary neutrophil accumulation. Data were expressed as means ± S. E. M. N ≥ 3 mice for each group. ** and *** indicated statistically significant difference-*p* < 0.01 and *p* < 0.001, respectively.

A recent report shows that Ly-GDI expression significantly inhibits FcγR-mediated phagocytosis [1], however, its role in inflammation mediated by FcγR activation is unknown. We determined Ly-GDI expression in IgG IC-injured lung. As shown in Fig 1, lung Ly-GDI expression assessed by real-time PCR (Fig 1A) and Western blot (Fig 1B) was significantly decreased 4 h after IgG IC deposition in the lung. To evaluate the role of Ly-GDI in FcγR-mediated acute lung inflammation, we constructed recombinant adenovirus which could express Ly-GDI with a Flag tag in its N-terminal region (Adeno-Ly-GDI). *In vitro* experiment demonstrated that Ly-GDI expression was dramatically elevated in HEK293 cells infected by Adeno-Ly-GDI when compared with cells incubated with control virus (Adeno-GFP, Fig 2A). To further determine if Adeno-Ly-GDI could mediate Ly-GDI expression in mouse lungs, we intratracheally administrated different doses of Adeno-Ly-GDI (2.5×10^8^ and 5 × 10^8^ PFU) into mouse lung. 72 h later, whole lung homogenates were used to perform Western blot to examine Ly-GDI expression. As shown in Fig 2B, adenovirus-mediated Ly-GDI expression was evident at a dose of 5×10^8^ PFU, and no strong Ly-GDI expression could not be observed in Adeno-GFP- and low dose Adeno-Ly-GDI-infected mouse lungs.

Next, we sought to determine the role of Ly-GDI in IgG IC-induced acute lung inflammation. 4 h after alveolar deposition of IgG IC, cell-free BAL fluids were harvested. Mouse albumin level in the BAL fluids was measured by ELISA, and permeability index was expressed as a ratio of total albumin in BAL fluid obtained from the inflamed lung to an average albumin content of the corresponding control group. We found that IgG IC treatment resulted in a marked increase in permeability index (Fig 2C). Notably, permeability index was greatly reduced in mice receiving airway administration of Adeno-Ly-GDI when compared with mice that received Adeno-GFP (69%, *p*<0.001, Fig 2C). Next, we measured lung MPO activity, which is a marker for neutrophil accumulation. As shown in Fig 2D, MPO activity in IgG IC-injured lung was dramatically elevated compared with that of control mice receiving anti-BSA only. Consistent with permeability index, IgG IC-induced lung MPO activity was significantly lower in mice receiving Adeno-Ly-GDI when compared with control counterparts receiving Adeno-GFP (32%, *p*<0.01, Fig 2D). These data together indicate that Ly-GDI suppressed pulmonary capillary leakage and neutrophil recruitment during IgG IC-induced acute lung injury.

**Fig 3:**
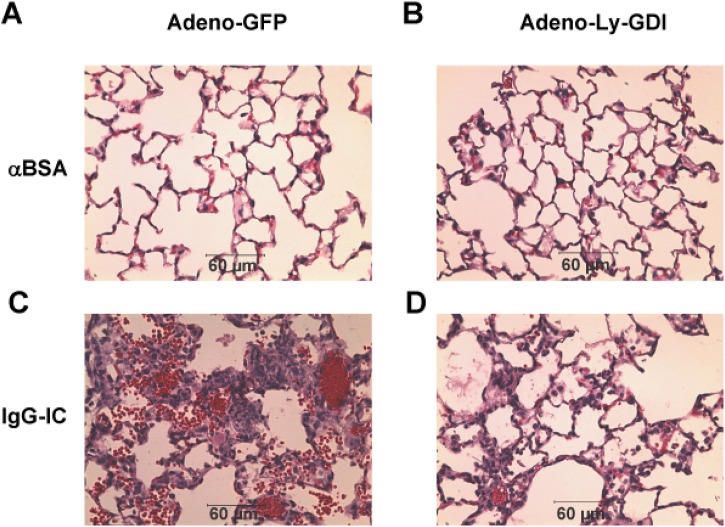
Histologically, Ly-GDI expression attenuates IgG immune complex-induced acute lung inflammation. Adeno-GFP or Adeno-Ly-GDI was injected to mice *via* an airway instillation at a dose of 5 × 10^8^ PFU. 3 days later, the mice were subjected to IgG immune complex-induced lung injury or control treatment (αBSA only). Lungs were harvested 4 h later, and lung sections were stained with H & E (40 × magnification). Lung sections shown included: Adno-GFP + anti-BSA (A), Adeno-GFP + IgG IC (B), Adeno-Ly-GDI + anti-BSA (C), and Adeno-Ly-GDI + IgG IC (D). Scale bar, 60 μm.

We further examined if mice that received intratracheal administration of Adeno-Ly-GDI exhibited attenuated lung injury by histological assay. We observed that airway injection of Adeno-GFP or Adeno-Ly-GDI had no influence on pulmonary inflammation and normal lung architecture (Fig 3A and 3C). Mice received Adeno-Ly-GDI together with IgG IC exhibited lung edema, hemorrhage, and neutrophil accumulation (Fig 3B). In contrast, all of these features were greatly attenuated in mice receiving Adeno-Ly-GDI during IgG IC-induced acute lung inflammation (Fig 3D).

### IgG IC-Induced Leukocyte Accumulation in the Alveoli Is Decreased by Ly-GDI

**Fig 4:**
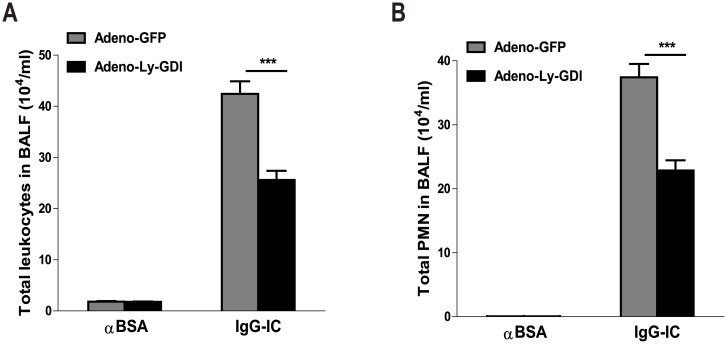
Over-expression of Ly-GDI reduces IgG immune complex-stimulated leukocytes accumulation in alveoli. Adeno-GFP or Adeno-Ly-GDI was injected to mice *via* an airway instillation at a dose of 5 × 10^8^ PFU. 3 days later, the mice were subjected to IgG immune complex-induced lung injury or control treatment (αBSA only). Total leukocytes **(A)** and neutrophils **(B)** in BAL fluids were counted 4 h later. Data were expressed as means ± S. E. M. N ≥ 3 mice for each group. *** indicated statistically significant difference-*p* < 0.001.

We determined the total leukocyte and neutrophil contents in BAL fluids. As shown in Fig 4A and 4h after IgG IC deposition in the lung, the number of leukocytes present in BAL fluids was significantly increased as compared with control mice challenged with anti-BSA alone. In injured lungs, the major cells recovered from BAL fluids were neutrophils (Fig 4). Importantly, we found that in the presence of IgG IC, mice receiving Adeno-Ly-GDI showed a significant decrease of total leukocytes and neutrophil accumulation in airspaces when compared with control mice receiving Adeno-GFP (40%, *p*<0.001 and 39%, *p*<0.001, respectively, Fig 4). Thus, Ly-GDI inhibited migration of leukocytes, especially neutrophils into the alveoli in IgG IC-injured mice.

### Ly-GDI Regulates Cytokine and Chemokine Expression in the Lung Challenged by IgG IC

**Fig 5:**
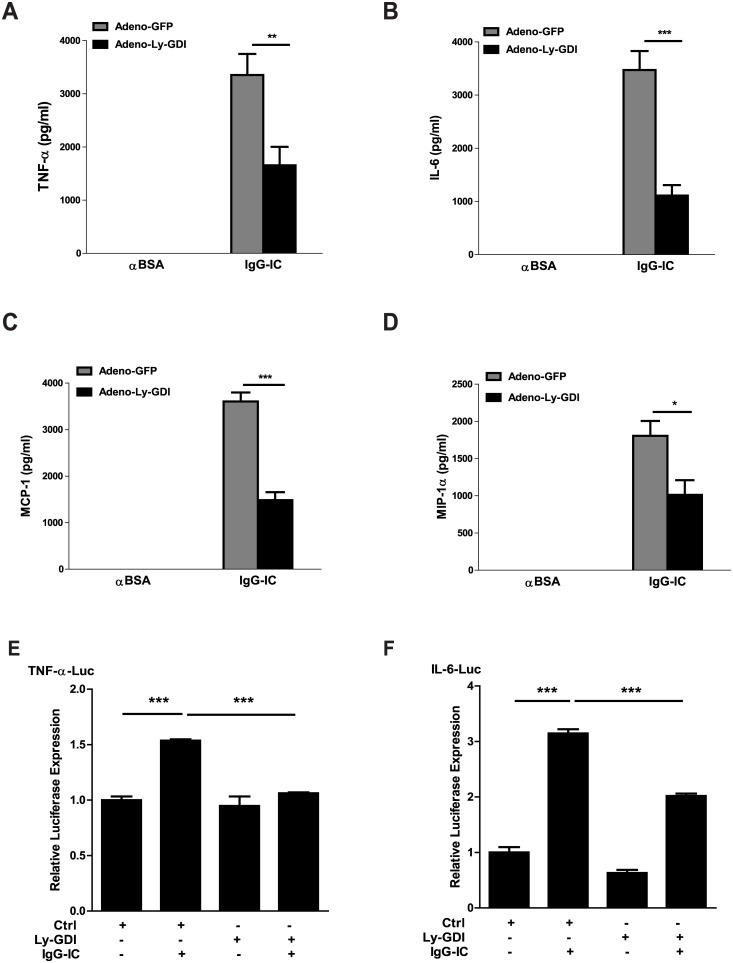
Over-expression of Ly-GDI decreases IgG immune complex-induced generation of pro-inflammatory mediators in BAL fluids. **(A-D)** Adeno-GFP or Adeno-Ly-GDI was injected to mice *via* an airway instillation at a dose of 5 × 10^8^ PFU. 3 days later, the mice were subjected to IgG immune complex-induced lung injury or control treatment (αBSA only). Cell-free BAL fluids were harvested 4 h later, and ELISAs were conducted to measure the TNF-α **(A)**, IL-6 **(B)**, MCP-1 **(C)**, and MIP-1α **(D)** levels. **(E and F)** MH-S cells were transiently transfected with indicated genes. 48 h later, the cells were treated with or without 100 μg/ml IgG immune complexes for 4 h. The cell lysates were used to evaluate luciferase expression. Luminometer values were normalized for expression from a co-transfected thymidine kinase reporter gene. These values were also normalized to a relative value of 1 for the control plasmid-transfected cells that did not receive IgG immune complex stimulation. Data were expressed as means ± S. E. M. N ≥ 3 mice for each group. *, ** and *** indicated statistically significant difference- *p<0*.*05*, *p* < 0.01 and *p* < 0.001, respectively.

We evaluated the role of Ly-GDI expression in inflammatory cytokine and chemokine generation in BAL fluids 4 h after pulmonary deposition of IgG IC. As shown in Fig 5, intratracheal administration of adenovirus alone without IgG IC challenge could not induce an increase in the levels of the indicated proinflammatory mediators. BAL fluids from IgG IC-injured mice receiving Adeno-GFP or Adeno-Ly-GDI exhibited dramatically augmented expressions of TNF-α, IL-6, and CC chemokines-MCP-1 and MIP-1α (Fig 5). Moreover, in IgG IC-injured lungs, ectopic expression of Ly-GDI led to a significant reduced production of TNF-α, IL-6, MCP-1, and MIP-1α (by 51%, 68%, 71%, and 44%, respectively) when compared with mice receiving airway administration of Adeno-GFP (Fig 5). However, there was no statistical difference in MIP-1β, KC, and MIP-2 production between Adeno-GFP-treated mice and Adeno-Ly-GDI-treaed mice in the presence of IgG IC (data not shown). These data suggest that Ly-GDI selectively inhibited certain pro-inflammatory mediators’ production during IgG IC-induced acute lung injury.

To further determine the effect of Ly-GDI on IgG IC-stimulated inflammation, we assessed whether Ly-GDI affected luciferase expressions controlled by IL-6 promoter and TNF-α promoter, respectively, in alveolar macrophage, MH-S cells, incubated in the presence or absence of IgG IC. As shown in Fig 5E and 5F, both IL-6- and TNF-luciferase expression were significantly augmented by IgG IC stimulation. Notably, over-expression of Ly-GDI dramatically suppressed the IgG IC-induced luciferase activities in MH-S cells.

### Ly-GDI Plays a Negative Role in C5a Level in BAL Fluids Recovered from IgG IC-Injured Lungs

**Fig 6:**
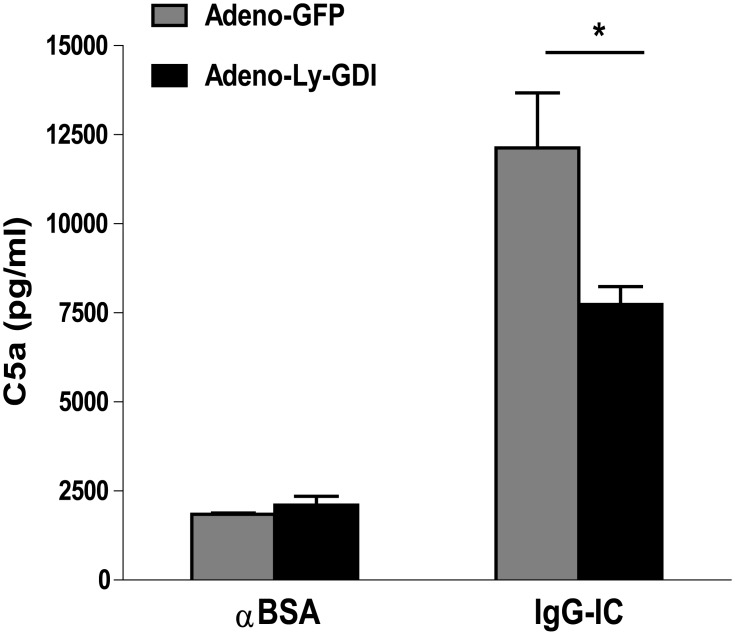
Ly-GDI over-expression leads to reduced IgG immune complex-induced production of C5a in BAL fluids. Adeno-GFP or Adeno-Ly-GDI was injected to mice *via* an airway instillation at a dose of 5 × 10^8^ PFU. 3 days later, the mice were subjected to IgG immune complex-induced lung injury or control treatment (αBSA only). Cell-free BAL fluids were harvested 4 h later, and ELISA was conducted to measure the C5a content. Data were expressed as means ± S. E. M. N ≥ 3 mice for each group. * indicated statistically significant difference- *p* < 0.05.

Previous study has shown that C5a is required for the full production of TNF-α, pulmonary neutrophil accumulation, and lung injury in IgG IC-induced acute lung inflammation model [19]. Therefore, we sought to elucidate whether C5a level in BAL fluids could be regulated by Ly-GDI. We found that Adeno-Ly-GDI alone without IgG IC treatment resulted in a moderate increase in the BAL fluid C5a level as compared with Adeno-GFP treatment, but there was no statistical difference (Fig 6). Moreover, there was a significant increase in C5a content in BAL fluids obtained from IgG IC-inflamed lungs (Fig 6). Notably, in the presence of IgG IC deposition, Adeno-Ly-GDI mice showed a significantly reduced C5a level in BAL fluids when compared with Adeno-GFP mice (Fig 6).

### Ly-GDI Inhibits IgG IC-Induced NF-κB Activity in the Lung and Alveolar Macrophages

**Fig 7:**
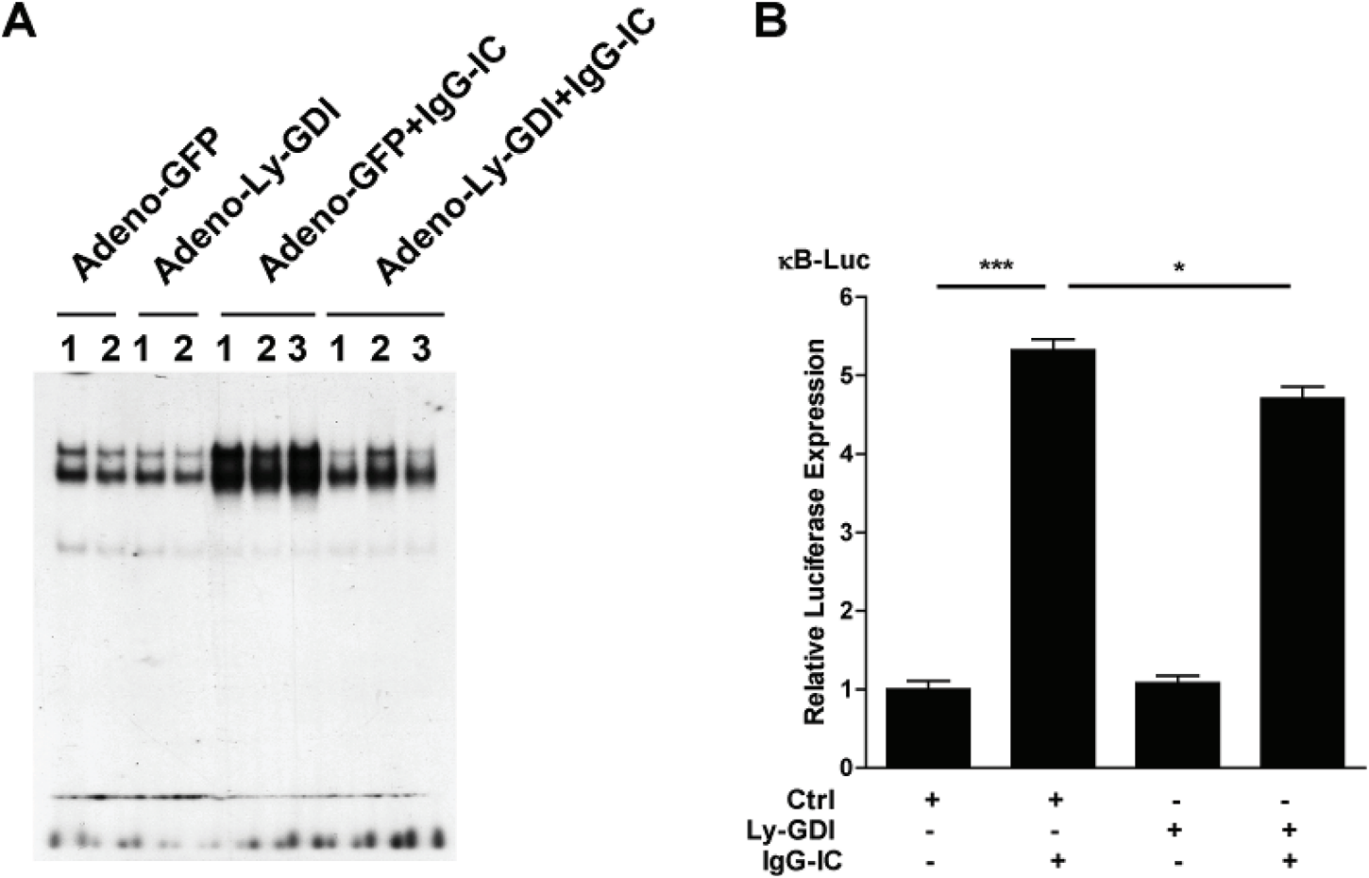
Ly-GDI inhibits IgG immune complex-induced NF-κB activity in the lung and alveolar macrophages. **(A)** Adeno-GFP or Adeno-Ly-GDI was injected to mice *via* an airway instillation at a dose of 5 × 10^8^ PFU. 3 days later, the mice were subjected to IgG immune complex-induced lung injury or control treatment (αBSA only). Lungs were harvested 4 h later. Whole lung nuclear proteins were extracted, and subjected to EMSA to determine the NF-κB DNA binding activity. **(B)** MH-S cells were transiently transfected with indicated genes. 48 h later, the cells were treated with or without 100 μg/ml IgG immune complexes for 4 h. The cell lysates were used to evaluate luciferase expression. Luminometer values were normalized for expression from a co-transfected thymidine kinase reporter gene. These values were then normalized to a relative value of 1 for the control plasmid transfected cells that did not receive IgG IC challenge. The data were expressed as means ± S. E. M. N = 3. * and *** indicated statistically significant difference-*p* < 0.05, and *p* < 0.001, respectively.

We sought to determine the potential mechanism by which Ly-GDI mitigated IgG-IC-induced inflammation in the lung. We infected mice with Adeno-GFP or Adeno-Ly-GDI by airway injection. 3 d later, the mice were challenged with or without IgG IC for 4 h, and the whole lung nuclear extracts were subjected to EMSA. As shown in Fig 7A, IgG IC significantly induced NF-κB DNA binding activity when compared with anti-BSA challenge alone. Notably, the amount of lung NF-κB associated with its consensus sequence was markedly reduced in IgG IC-inflamed mice receiving airway administration of Adeno-Ly-GDI when compared with their littermates intratracheally injected with Adeno-GFP (Fig 7A). Alveolar macrophages contributed to full activation of NF-κB in IgG IC-induced acute lung inflammation model [23]. To elucidate the role of Ly-GDI in IgG IC-induced NF-κB activation in alveolar macrophages, we performed *in vitro* luciferase experiments by using MH-S cells. As shown in Fig 7B, IgG IC treatment led to an over 5-fold increase of luciferase production driven by NF-κB, and over-expression of Ly-GDI caused a significant decrease of luciferase expression in MH-S cells.

## Discussion

Ly-GDI suppresses the activation of small GTPases of the Rho family by inhibiting dissociation of GDP from Rho proteins. Although the Rho family plays critical roles in inflammatory signal transduction cascades, however, the function of Ly-GDI in immune system remains largely unknown. Ly-GDI was originally identified as a metastasis suppressor in bladder cancer during studies of the differential invasive and metastatic properties of isogenic human bladder carcinoma cell lines [24]. Using Ly-GDI knock-out mice, Yin et al show that Ly-GDI is not essential for development of the immune system or many immunological functions, but significantly influences lymphocyte growth and survival during *in vitro* cultivation [25]. In addition, Ly-GDI associates with CARD9, a caspase-recruitment domain (CARD)-containing protein, in phagosomes after bacterial and fungal infection, and binding of CARD9 suppressed LyGDI-mediated inhibition of the GTPase Rac1, thereby leading to bacterial killing in macrophages [26]. Ectopic expression of Ly-GDI greatly inhibited FcγR-mediated phagocytosis in human monocytes, but its role in FcγR activation-triggered inflammatory reactions was still unknown [1]. In the current report, we show that Ly-GDI expression was significantly reduced in lungs during IgG IC-induced ALI. Importantly, Ly-GDI plays a negative role in IgG IC-induced inflammatory responses in the lung and alveolar macrophages.

During IgG IC-induced ALI, alveolar macrophages are activated, and play a critical role in IgG IC-stimulated generation of pro-inflammatory mediators including a variety of chemokines and cytokines [27]. For example, production of TNF-α that is considered as an early response cytokine was dramatically increased in IgG IC-injured lungs, which contributed to the elevated synthesis of chemokines such as MIP-1α, and final pulmonary injury including increased lung microvascular permeability, neutrophil accumulation, and pulmonary hemorrhage [17, 19, 20, 28, 29]. MIP-1α belongs to CC chemokine family, and is required for formation of chemoattractant gradients and subsequent transmigration of polymorphonuclear neutrophils (PMNs) from vascular vessels into lungs [30]. MCP-1 is also a member of CC chemokine family, and plays an essential role in regulating macrophage infiltration. Our finding that over-expression of Ly-GDI led to a decreased expression of various pro-inflammatory mediators including TNF-α, IL-6, MCP-1, and MIP-1α suggests that Ly-GDI regulates IgG IC-induced ALI by cytokine and chemokine pathways. In addition, the reduced MCP-1 production may contribute to decreased accumulation of macrophages in lungs, which further leads to inhibition of IgG IC-induced lung inflammation and injury. Interestingly, a recent study implicates that Ly-GDI suppresses cancer metastasis by reducing MCP-1 expression in tumor microenvironment, which was required for macrophage infiltration [31].

The molecular mechanism by which Ly-GDI affects cytokine and chemokine gene expression is not clear. RhoA, Rac, and Cdc42 all participate in the signaling pathways required for the activity of NF-κB, leading to the induction of cytokines and chemokines [32, 33]. Furthermore, the activation of NF-κB has been linked to Rho-dependent activation of the c-Jun NH2-terminal kinase/stress-activated protein kinase pathway [32, 34]. Our current study shows that Ly-GDI reduces the activation of both lung NF-κB (Fig 7) and Stat3 (data not shown). These two transcription factors are important regulators of inflammatory mediators. Thus, it is possible that Ly-GDI mitigates IgG IC-induced cytokine and chemokine expression by regulating NF-κB and Stat3 activity in the lung. However, the detailed pathways for this regulation remain to be determined.

Another interesting finding in this study is that C5a generation in BAL fluids was significantly reduced in mice receiving Adeno-Ly-GDI when compared with control mice treated with Adeno-GFP during IgG IC-induced ALI. Our previous studies showed that pulmonary STAT3 was activated after airway deposition of IgG IC, which positively regulated IgG IC-induced acute pulmonary inflammation [15, 18]. In addition, STAT3 activation was dependent on C5a/C5aR signaling pathway in lungs challenged by IgG IC [15]. Thus, Ly-GDI-mediated C5a reduction may lead to STAT3 inhibition in IgG IC-injured lung.

Fcγ receptors (FcγRs), which bind to the Fc portion of IgG and induce inflammatory reactions, play essential roles in immune defense system. The roles of FcγRs in IgG IC-induced autoimmune diseases are extensively investigated, but growing evidences suggest that they also play an important role in the regulation of innate immunity. For example, it was recently shown that binding of IgG IC that is formed by interaction between peptidoglycan (PGN) and the corresponding IgG antibody to FcγRIIA was indispensible for PGN-stimulated inflammation [35]. Because PGN is a major structural component of Gram-positive bacteria, these data suggest that FcγRIIA may be involved in Gram-positive bacteria-mediated systemic inflammatory responses [35]. This is supported by the finding that high expression of FcγRIIA on human neutrophils is a sepsis biomarker [36, 37]. Moreover, there may be a crosstalk between FcγRs and Toll-like receptors (TLRs) signaling pathways. For example, IgG IC could stimulate interaction between TLR4 and FcγRIII, and disruption of the association by knockout or mutation of TLR4 led to decreased inflammatory responses triggered by IgG IC [38]. Interestingly, TLR4-induced TRIF-dependent pathway was positively regulated by FcγIIA, whereas TLR4-triggered MyD88-dependent signaling pathway was suppressed by FcγIIA in human monocytes [39]. Here, we showed that over-expression of Ly-GDI resulted in inhibition of IgG IC-induced NF-κB transcriptional activity; however, whether Ly-GDI could regulate TRIF/MyD88 pathways or suppress the interaction between TLR4 and FcγRs to affect NF-κB remains an open question. Taken together, these studies and our current finding that Ly-GDI is an important regulator of lung inflammatory injury after deposition of IgG IC may help to understand a new signaling pathway which is important for both inflammation/infection and autoimmune diseases.

## References

[1] Mehta P, Wavreille AS, Justiniano SE, Marsh RL, Yu J, Burry RW, et al LyGDI, a novel SHIP-interacting protein, is a negative regulator of FcgammaR-mediated phagocytosis. PLoS One. 6(6):e21175 10.1371/journal.pone.0021175 21695085 PMC3114867

[2] Dovas A, Couchman JR. RhoGDI: multiple functions in the regulation of Rho family GTPase activities. Biochem J. 2005;390(Pt 1):1–9. . 16083425 10.1042/BJ20050104 PMC1184558

[3] Lelias JM, Adra CN, Wulf GM, Guillemot JC, Khagad M, Caput D, et al cDNA cloning of a human mRNA preferentially expressed in hematopoietic cells and with homology to a GDP-dissociation inhibitor for the rho GTP-binding proteins. Proc Natl Acad Sci U S A. 1993;90(4):1479–83. . 8434008 10.1073/pnas.90.4.1479 PMC45897

[4] Bishop AL, Hall A. Rho GTPases and their effector proteins. Biochem J. 2000;348 Pt 2:241–55. . 10816416 PMC1221060

[5] Scherle P, Behrens T, Staudt LM. Ly-GDI, a GDP-dissociation inhibitor of the RhoA GTP-binding protein, is expressed preferentially in lymphocytes. Proc Natl Acad Sci U S A. 1993;90(16):7568–72. . 8356058 10.1073/pnas.90.16.7568 PMC47183

[6] Leffers H, Nielsen MS, Andersen AH, Honore B, Madsen P, Vandekerckhove J, et al Identification of two human Rho GDP dissociation inhibitor proteins whose overexpression leads to disruption of the actin cytoskeleton. Exp Cell Res. 1993;209(2):165–74. . 8262133 10.1006/excr.1993.1298

[7] Groysman M, Hornstein I, Alcover A, Katzav S. Vav1 and Ly-GDI two regulators of Rho GTPases, function cooperatively as signal transducers in T cell antigen receptor-induced pathways. J Biol Chem. 2002;277(51):50121–30. 10.1074/jbc.M204299200 . 12386169

[8] Robles-Carrillo L, Meyer T, Hatfield M, Desai H, Davila M, Langer F, et al Anti-CD40L immune complexes potently activate platelets in vitro and cause thrombosis in FCGR2A transgenic mice. J Immunol. 185(3):1577–83. 10.4049/jimmunol.0903888 20585032

[9] Smith KG, Clatworthy MR. FcgammaRIIB in autoimmunity and infection: evolutionary and therapeutic implications. Nat Rev Immunol. 10(5):328–43. 10.1038/nri2762 20414206 PMC4148599

[10] Schuyler M, Gott K, French V. The role of MIP-1alpha in experimental hypersensitivity pneumonitis. Lung. 2004;182(3):135–49. . 15526753 10.1007/s00408-004-0311-7

[11] Schaller M, Burton DR, Ditzel HJ. Autoantibodies to GPI in rheumatoid arthritis: linkage between an animal model and human disease. Nat Immunol. 2001;2(8):746–53. . 11477412 10.1038/90696

[12] Nimmerjahn F, Ravetch JV. FcgammaRs in health and disease. Curr Top Microbiol Immunol. 2011;350:105–25. 10.1007/82_2010_86 . 20680807

[13] Nimmerjahn F, Ravetch JV. Fcgamma receptors as regulators of immune responses. Nat Rev Immunol. 2008;8(1):34–47. 10.1038/nri2206 . 18064051

[14] Huber-Lang M, Sarma JV, Zetoune FS, Rittirsch D, Neff TA, McGuire SR, et al Generation of C5a in the absence of C3: a new complement activation pathway. Nat Med. 2006;12(6):682–7. . 16715088 10.1038/nm1419

[15] Gao H, Guo RF, Speyer CL, Reuben J, Neff TA, Hoesel LM, et al Stat3 activation in acute lung injury. J Immunol. 2004;172(12):7703–12. . 15187153 10.4049/jimmunol.172.12.7703

[16] Gao H, Hoesel LM, Guo RF, Rancilio NJ, Sarma JV, Ward PA. Adenoviral-mediated overexpression of SOCS3 enhances IgG immune complex-induced acute lung injury. J Immunol. 2006;177(1):612–20. . 16785559 10.4049/jimmunol.177.1.612

[17] Warren JS, Yabroff KR, Remick DG, Kunkel SL, Chensue SW, Kunkel RG, et al Tumor necrosis factor participates in the pathogenesis of acute immune complex alveolitis in the rat. J Clin Invest. 1989;84(6):1873–82. . 2531759 10.1172/JCI114374 PMC304067

[18] Tang H, Yan C, Cao J, Sarma JV, Haura EB, Wu M, et al An essential role for Stat3 in regulating IgG immune complex-induced pulmonary inflammation. Faseb J. 25(12):4292–300. 10.1096/fj.11-187955 21859893 PMC3236634

[19] Czermak BJ, Sarma V, Bless NM, Schmal H, Friedl HP, Ward PA. In vitro and in vivo dependency of chemokine generation on C5a and TNF-alpha. J Immunol. 1999;162(4):2321–5. . 9973510

[20] Guo RF, Ward PA. Mediators and regulation of neutrophil accumulation in inflammatory responses in lung: insights from the IgG immune complex model. Free Radic Biol Med. 2002;33(3):303–10. . 12126752 10.1016/s0891-5849(02)00823-7

[21] Yan C, Wu M, Cao J, Tang H, Zhu M, Johnson PF, et al Critical role for CCAAT/enhancer-binding protein beta in immune complex-induced acute lung injury. J Immunol. 189(3):1480–90. 10.4049/jimmunol.1200877 22732594 PMC3401325

[22] Yan C, Johnson PF, Tang H, Ye Y, Wu M, Gao H. CCAAT/Enhancer-Binding Protein delta Is a Critical Mediator of Lipopolysaccharide-Induced Acute Lung Injury. Am J Pathol. . 23177475 10.1016/j.ajpath.2012.10.013 PMC3562738

[23] Lentsch AB, Czermak BJ, Bless NM, Van Rooijen N, Ward PA. Essential role of alveolar macrophages in intrapulmonary activation of NF-kappaB. Am J Respir Cell Mol Biol. 1999;20(4):692–8. . 10101001 10.1165/ajrcmb.20.4.3414

[24] Said N, Theodorescu D. Pathways of metastasis suppression in bladder cancer. Cancer Metastasis Rev. 2009;28(3–4):327–33. 10.1007/s10555-009-9197-4 . 20013033

[25] Yin L, Schwartzberg P, Scharton-Kersten TM, Staudt L, Lenardo M. Immune responses in mice deficient in Ly-GDI, a lymphoid-specific regulator of Rho GTPases. Mol Immunol. 1997;34(6):481–91. . 9307064 10.1016/s0161-5890(97)00053-9

[26] Wu W, Hsu YM, Bi L, Songyang Z, Lin X. CARD9 facilitates microbe-elicited production of reactive oxygen species by regulating the LyGDI-Rac1 complex. Nat Immunol. 2009;10(11):1208–14. 10.1038/ni.1788 19767757

[27] Gao H, Neff T, Ward PA. Regulation of lung inflammation in the model of IgG immune-complex injury. Annu Rev Pathol. 2006;1:215–42. . 18039114 10.1146/annurev.pathol.1.110304.100155

[28] Lentsch AB, Czermak BJ, Bless NM, Ward PA. NF-kappaB activation during IgG immune complex-induced lung injury: requirements for TNF-alpha and IL-1beta but not complement. Am J Pathol. 1998;152(5):1327–36. . 9588901 PMC1858598

[29] Chouchakova N, Skokowa J, Baumann U, Tschernig T, Philippens KM, Nieswandt B, et al Fc gamma RIII-mediated production of TNF-alpha induces immune complex alveolitis independently of CXC chemokine generation. J Immunol. 2001;166(8):5193–200. . 11290803 10.4049/jimmunol.166.8.5193

[30] Shanley TP, Schmal H, Friedl HP, Jones ML, Ward PA. Role of macrophage inflammatory protein-1 alpha (MIP-1 alpha) in acute lung injury in rats. J Immunol. 1995;154(9):4793–802. . 7722328

[31] Said N, Sanchez-Carbayo M, Smith SC, Theodorescu D. RhoGDI2 suppresses lung metastasis in mice by reducing tumor versican expression and macrophage infiltration. J Clin Invest. 122(4):1503–18. 10.1172/JCI61392 22406535 PMC3314474

[32] Montaner S, Perona R, Saniger L, Lacal JC. Multiple signalling pathways lead to the activation of the nuclear factor kappaB by the Rho family of GTPases. J Biol Chem. 1998;273(21):12779–85. . 9582304 10.1074/jbc.273.21.12779

[33] Cordle A, Koenigsknecht-Talboo J, Wilkinson B, Limpert A, Landreth G. Mechanisms of statin-mediated inhibition of small G-protein function. J Biol Chem. 2005;280(40):34202–9. 10.1074/jbc.M505268200 . 16085653

[34] Coso OA, Chiariello M, Yu JC, Teramoto H, Crespo P, Xu N, et al The small GTP-binding proteins Rac1 and Cdc42 regulate the activity of the JNK/SAPK signaling pathway. Cell. 1995;81(7):1137–46. . 7600581 10.1016/s0092-8674(05)80018-2

[35] Sun D, Raisley B, Langer M, Iyer JK, Vedham V, Ballard JL, et al Anti-peptidoglycan antibodies and Fcgamma receptors are the key mediators of inflammation in Gram-positive sepsis. J Immunol. 189(5):2423–31. 10.4049/jimmunol.1201302 22815288 PMC3424298

[36] Hoffmann JJ. Neutrophil CD64 as a sepsis biomarker. Biochem Med (Zagreb). 21(3):282–90. . 22420242 10.11613/bm.2011.038

[37] Hsu KH, Chan MC, Wang JM, Lin LY, Wu CL. Comparison of Fcgamma receptor expression on neutrophils with procalcitonin for the diagnosis of sepsis in critically ill patients. Respirology. 16(1):152–60. 10.1111/j.1440-1843.2010.01876.x 20946336

[38] Rittirsch D, Flierl MA, Day DE, Nadeau BA, Zetoune FS, Sarma JV, et al Cross-talk between TLR4 and FcgammaReceptorIII (CD16) pathways. PLoS Pathog. 2009;5(6):e1000464 10.1371/journal.ppat.1000464 19503602 PMC2685003

[39] Shalova IN, Kajiji T, Lim JY, Gomez-Pina V, Fernandez-Ruiz I, Arnalich F, et al CD16 regulates TRIF-dependent TLR4 response in human monocytes and their subsets. J Immunol. 188(8):3584–93. 10.4049/jimmunol.1100244 22427642

